# Load-bearing capacity of an experimental dental implant made of Nb-1Zr

**DOI:** 10.1007/s10856-025-06858-7

**Published:** 2025-01-15

**Authors:** Philipp-Cornelius Pott, Karolina Petsa, Christian Klose, Julian-Tobias Schleich, Neele Brümmer, Andreas Winkel, Hans Jürgen Maier, Meike Stiesch

**Affiliations:** 1 Clinic of Prosthetic Dentistry and Biomedical Materials Research, Hannover Medical School, Hannover, Germany; 2 Institut für Werkstoffkunde (Materials Science), Leibniz University Hannover, An der Universität 2, Garbsen, Germany

## Abstract

Although implants have undergone a remarkable development over the past decades, modern implants still show complications that make the improvement of materials necessary. The presented study investigates the load-bearing capacity of an experimental dental implant made of a niobium alloy (Nb1Zr) compared to identical implants made of Ti6Al4V using chewing simulation for artificial aging. Eight implants each with an experimental design were manufactured from Nb1Zr and Ti6Al4V. A standardized abutment made of Ti6Al4V was fabricated for each implant and screwed into the implant with a screw made of the same material. A shape-identical crown for an upper first molar was fabricated for all implants using the CAD/CAM technique. All specimens were artificially aged using chewing simulation for 1 × 10^6^ cycles and thermocycling between 5 °C and 55 °C for 4 × 10^3^ cycles. After that, all specimens were loaded until failure. This was followed by a 3D analysis of the deformation of the samples. 100% of the samples survived the artificial aging. The Nb1Zr samples failed at 2595 ± 1069 N. In the Ti6Al4V group, failure occurred at 2958 ± 1058 N. The first deformations occurred in both groups from a load of at least 1114 N. The 3D analysis revealed deformations of 0.08 mm in the implant shoulder area of the Nb1Zr implants and of 0.04 mm in the Ti6Al4V implants. This difference was significant (*p* = 0.016). The investigated Nb1Zr alloy appears to be suitable for implants. The measured load-bearing capacity is significantly higher than the expected intraoral masticatory forces.

**Graphical Abstract:**

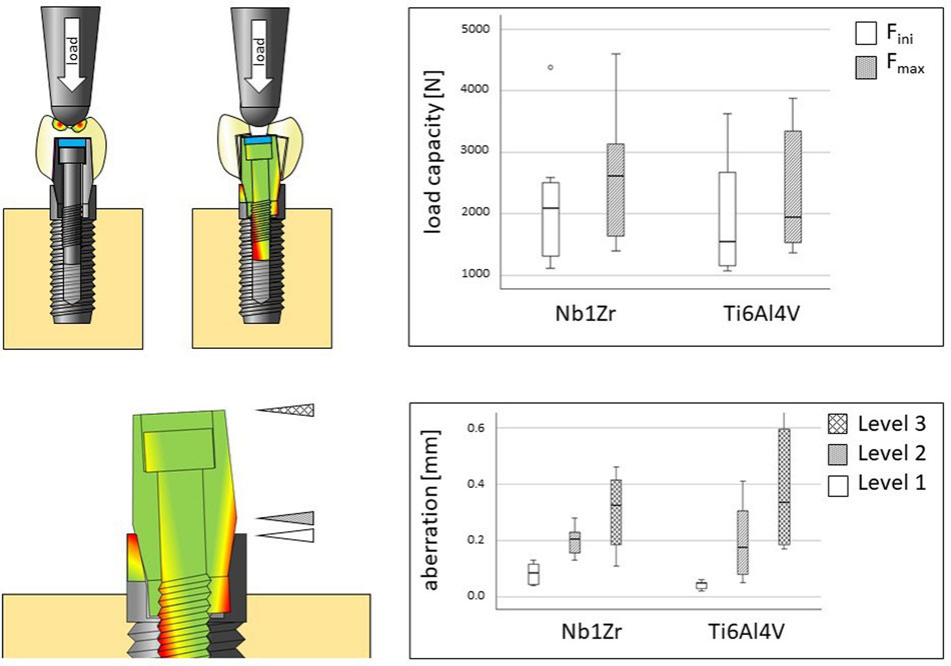

## Introduction

In modern dentistry, implants are an indispensable component of patient care, both with removable dentures and with fixed restorations. Mostly, the components of multi-part implant systems are made of the titanium alloy Ti6Al4V. In rare cases, there are allergic reactions with components of the Ti6Al4V alloy [1]. As an alternative multi-part implant systems made of high-performance ceramics such as zirconia are available. Ceramic implants sometimes show mechanical complications. A conclusive assessment of ceramic implants suitability has not been possible yet, due to lack of data [2]. The need for alternative implant materials with high mechanical stability and the absence of the mentioned disadvantages remain. In this study, we present the niobium-based alloy Nb1Zr as a possible alternative. Niobium alloys are characterized by good mechanical strength, high biocompatibility and a high potential for osseointegration [3–5]. Nb1Zr does not contain any critical components such as aluminium [6]. Therefore, there are no allergically reactions to be expected. In its elementary form, niobium is not sufficiently stable for use as an implant material, which is why it is currently only used for coatings [3, 4]. The stability of niobium alloys can be increased without negatively affecting the other material properties when using suitable processing techniques. The ultra-fine grain in such alloys containing niobium results in excellent resistance to mechanical and chemical stresses [5, 7]. Therefore, the niobium-based alloy Nb1Zr should be explored as a possible alternative implant material.

In addition to mechanical complications, peri-implant inflammation in particular is a challenging complication with implant-supported restorations. According to Dreyer et al., up to 22% of implants develop peri-implant inflammation during the clinical use [8]. Such inflammations are triggered by bacterial biofilms and are initially limited to the marginal gingiva, as with natural teeth, but can then quickly proceed and affect the peri-implant bone if the biofilm persists. This leads to loss of hard and soft tissue and finally to the loss of the implant. In this context, high biocompatibility of the materials plays a decisive role. The attachment of tissue-forming cells should be as high as possible, while the adhesion of bacteria should be as low as possible. Niobium alloys are also promising in this respect. These alloys have already been certified to have good biocompatibility [3–5, 7]. By further improving the surface quality through the above-mentioned severe plastic deformation, an increase in biocompatibility can also be expected [5, 7]. In preliminary unpublished tests we showed that the bacterial colonization of a niobium alloy does not differ from the colonization of bacteria on Ti6Al4V. Further, the ability of fibroblasts to adhere on both alloys also was comparable to established materials. In this regard, further investigations will be carried out as part of the further development of the new experimental implant, which is described below.

Along the development of new implant materials completely novel implant designs are being explored. As part of the DFG-funded Collaborative Research Center TRR/SFB 599 SIIRI (Safety Integrated Infection Reactive Implants, Project-ID 426335750), we aim to develop an implant with an internal cavity for an antibacterial agent to be released through channels in the implant corpus and a porous implant surface coating into the surrounding tissue under the control of the dentist. The design of the implant body was developed at the Institute of Materials Science at Leibniz University Hannover in cooperation with the Clinic of Prosthetic Dentistry and Biomedical Materials Research at Hannover Medical School. In preliminary unpublished tests from the Institute of Materials Science, this implant geometry has already been examined in silico and in vitro in accordance with the ISO 14801 standard [9]. In view of the parameters mentioned above, niobium alloy appears to be a promising implant material to fulfill the needs for the clinical use and to withstand the human masticatory forces in the range between 150 N in healthy patients to 800 N, e.g. in case of bruxism.

The presented in-vitro study compares the load-bearing capacity of this experimental dental implant made of the niobium alloy Nb1Zr to implants of the same shape made of the titanium alloy Ti6Al4V using chewing simulation to mimik the clinical use. The following hypotheses were to be tested: (1) The implants made of Nb1Zr do not differ from implants made of Ti6Al4V in terms of load capacity. (2) The implants made of Nb1Zr can withstand loads above 800 N.

## Materials and methods

The load-bearing capacity of eight implants manufactured from both conventional coarse-grained Nb1Zr and Ti6Al4V was evaluated after chewing simulation. The implants were designed according to the concept of bone-level implants. The parameters described below were selected because implants of these dimensions can be used in most classical indications. The implant diameter is 4.5 mm, the length of the implant is 14 mm. The internal connection of the implant to the abutment is conical with an angle of 80° over a distance of 1.0 mm from the implant shoulder and then parallel-walled over a distance of 2.0 mm. The internal metric thread (M2) for the abutment screw has a diameter of 1.6 mm and extends 2.5 mm into the implant. Below this thread there is a 4.5 mm long cavity for the anti-infective agent. The implants were initially turned manually from material blanks produced by extrusion molding. The internal geometry was also manually machined into the implant blanks.

**Fig. 1:**
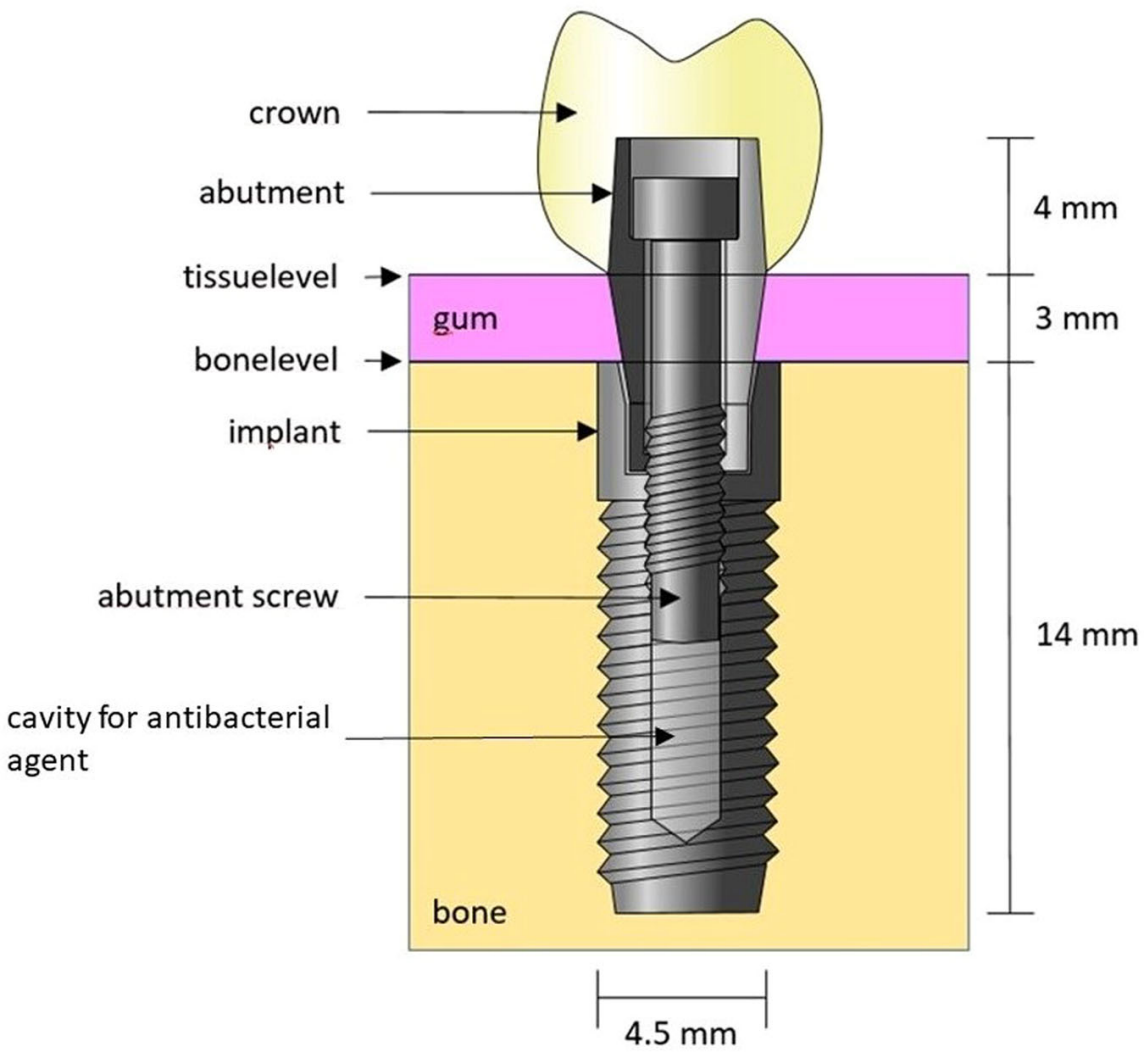
Schematic drawing of the experimental implant with the relevant components

The abutments for all of the implants were made of Ti6Al4V. A transmucosal height of 3 mm was taken into account when designing the abutments. The height of the retentive abutment surface is 4 mm (Fig. 1). The abutments were screwed into the implants using standardized cylindric screws made of Ti6Al4V (DIN 912/ISO 4762).

**Fig. 2:**
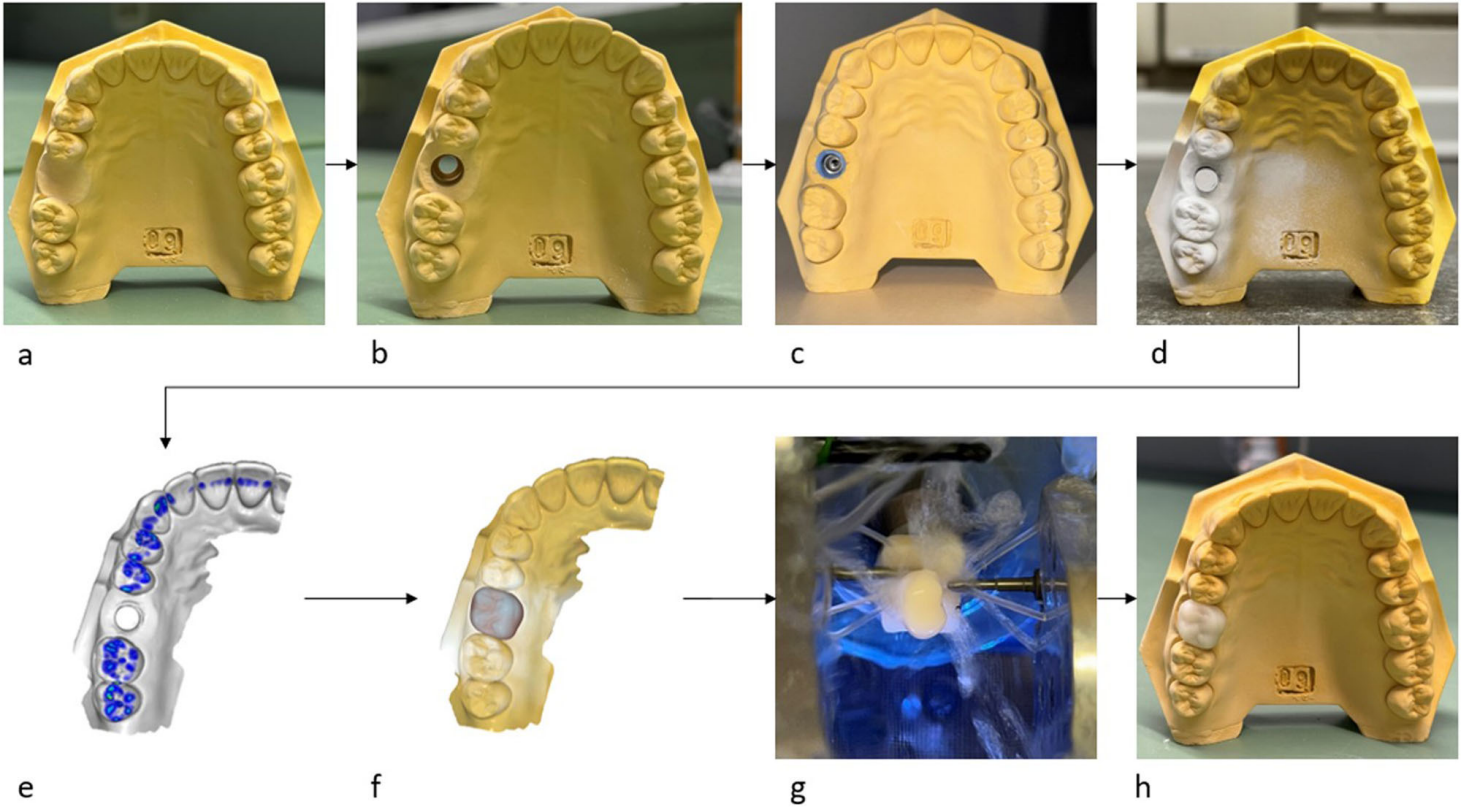
Manufacturing process of the prototype crown: Model without tooth 16 (**a**), prepared cavity for the implant (**b**), implant placed into the model (**c**), Implant-Abutment-System prepared for scanning (**d**), digital model of the situation (**e**), virtual crown-design (**f**), milling process (**g**), fitting of the prototype crown in the dental arch (**h**)

Identical zirconia crowns were fabricated for all specimens in a digital workflow. A standardized dental training model (Frasaco AG 3, Tettnang, Germany) served as the basis for the crown design. In the setting for this study, the tooth 16 was replaced with an implant crown. At first a copy of the model was made of plaster. This model with the original tooth 16 in the closed row of teeth was first digitized using a dental scanning system (Primescan, Dentsply Sirona, Bensheim, Germany). Next, the tooth 16 was removed from the model and a cavity for the implant placement was prepared simulating the clinical procedure. A prototype of the experimental implants was positioned into this cavity using putty silicone. The corresponding abutment was screwed into the implant and the situation was further digitized. The data sets were virtually superimposed. The outer contour of the model tooth 16, in particular its occlusal surface, was transferred to the digital design of the implant crown using a digital copy milling process (Cerec SW 5.4, Dentsply Sirona). A prototype crown was milled of IPS-Empress CAD (Ivoclar Vivadent GmbH, Ellwangen,Germany) to check the fit of the crown on the implant and in the dental arch (Fig. 2).

As the prototype crown met the requirements, a total of 16 crowns were produced from zirconia (Cercon HT, Dentsply Sirona) using a multiaxis milling machine (MCX-V, Dentsply Sirona). The crowns were sintered according to the manufacturer’s instructions (LHT 02/17, Nabertherm GmbH, Lilienthal, Germany). All crowns fitted directly without further adjustment on the abutments. The abutments were mounted into the implants using the titanium screws with a torque of 25 Ncm and the crowns were fixed temporarily onto the abutments using small amounts of adhesive wax on the crowns margins. Both for the chewing simulation and for the loading in an universal testing machine (UTS RetroLine, ZwickRoell GmbH & Co KG, Ulm, Germany), the test specimens had to be positioned in such a way that the applied force could act corresponding to the clinical situation. A ball made of stainless steel with a diameter of 6 mm was used to apply the force. The ball was positioned over the occlusal surface in such a way that contact resulted on the triangular ridges of the cusps when a load was applied, similar to a clinically ideal occlusal situation. A special apparatus developed at the ZPR-HMS using the identical geometry of the occlusal surfaces of all crowns ensured that all test specimens were embedded in an identical position in special specimen sockets using polyurethane. Care was taken to ensure that the polyurethane came within 2 mm of the implant shoulder. This ensured that, according to the previous in silico examinations [9], the area in which deformation of the implant could be expected was not supported by the polyurethane. After embedding, the crowns fixed with adhesive wax were detached from the abutments and all wax residue was removed.

According to the clinical procedure, the screw was covered with teflon to prevent the internal structures of the implant from getting filled up with cement material. The crowns were cemented to the abutments adhesively with SpeedCEM Plus (Ivoclar Vivadent GmbH, Ellwangen, Germany) in combination with Ceramic Bond following the manufacturer’s instructions (Voco GmbH, Cuxhaven, Germany). Excess cement was removed.

After initial storage in water at 36 °C, all samples underwent artificial aging in a chewing simulator (CS-4, SD-Mechatronik, Feldkirchen-Westerham, Germany) for 1 × 10^6^ cycles and 25 N load in combination with 4 × 10^3^ cycles of thermocycling between 5 °C and 55 °C during the chewing simulation. 24 h after artificial aging, the samples were loaded in a universal testing machine (UTS RetroLine, ZwickRoell GmbH & Co KG, Ulm, Germany) until failure. The tests were carried out with a preload of 2 N. Force until failure was protocolled in a force-distance diagram for every specimen. Failure was defined as fracture of the crown and/or a load drop of 20% of the maximum load (*F*_max_) achieved up to that point. The statistical comparisons between the two materials regarding the force at initial deformation (*F*_ini_) and the maximal load-bearing capacity (*F*_max_) was done by ANOVA and Tukey-HSD (IBM SPSS Statistics V29.0.1.0, IBM, Armonk, NY, USA). The level of significance was set to *p* = 0.05.

**Fig. 3:**
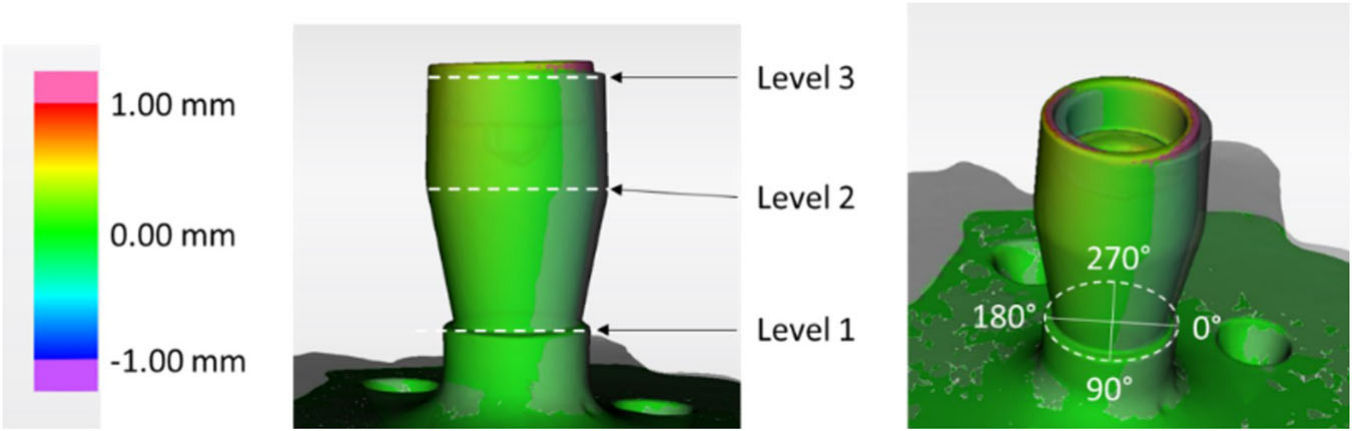
Position of the levels 1–3 for deviation measurements

Additionally, the abutments mounted into the embedded implants were digitized with the Primescan system at two points in time: First previous to cementation of the crowns onto the abutments for artificial aging and Second after removing the crowns or crown remnants following the stress test. To ensure the best-fit matching of these data sets, each specimen received individual markings in the polyurethane around the implant-abutment complex. Matching of these two data sets using special software for recording of three-dimensional volume deviations (OraCheck, Sirona, Bensheim, Germany) allowed a direct comparison of any deformations of the implant-abutment complex that may have occurred as a result of loading during artificial aging and the pressure load examination. To describe possible deviations, the measurements were done at three different levels: First – implant shoulder, Second – lowest coronal margin of the abutment, Third – highest coronal margin of the abutment. The software displays deviations between two data sets using a color-scale. The software also allows precise measurements between the two datasets by direct measuring of the distance between the digital surfaces on every single localization using a measuring cursor. The area of greatest deviation was identified for each test specimen and defined as the 0° position for all three levels. Starting from this position, three further measurements were carried out at 90°, 180° and 270° respectively. The deviations found between the data sets before and after loading were measured at these positions (Fig. 3). The statistical analysis of the deviations was also done by ANOVA and Tukey-HSD (IBM SPSS Statistics V29.0.1.0, IBM, Armonk, NY, USA). The level of significance was set to *p* = 0.05.

## Results

**Fig. 4:**
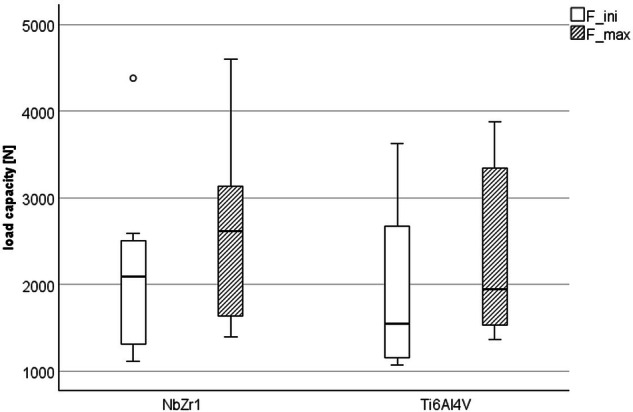
load-bearing capacity of the experimental implants made of Nb1Zr and Ti6Al4V, F_ini_ and F_max_ are given

We tested the novel Nb1Zr alloy in our test design and analyzed the deformation parameters. All specimens survived artificial aging by chewing simulation. In the subsequent compressive load testing, the NbZr1 samples showed initial deformation at *F*_ini_ = 2162.6 ± 1054.1 N and a maximal load (*F*_max_) of 2594.9 ± 1068.3 N. For the Ti6Al4V group lower mean values in *F*_ini_ (1929.2 ± 983.9 N) and *F*_max_ (2358.4 ± 1057.2 N) were measured (Fig. 4). However, the comparisons between the two materials of *F*_ini_ (*p* = 0.654) and *F*_max_ (*p* = 0.663) were statistically not significant.

In addition, total changes in length at *F*_max_ (*dL*_Fmax_) was measured for each sample during the load-bearing capacity test. *dL*_Fmax_ in the Nb1Zr group was 823 ± 262 µm, in the Ti6Al4V group *dL*_Fmax_ of 695 ± 130 µm was detected. There was no significant difference between the materials with regard to this parameter (*p* = 0.236).

**Fig. 5:**
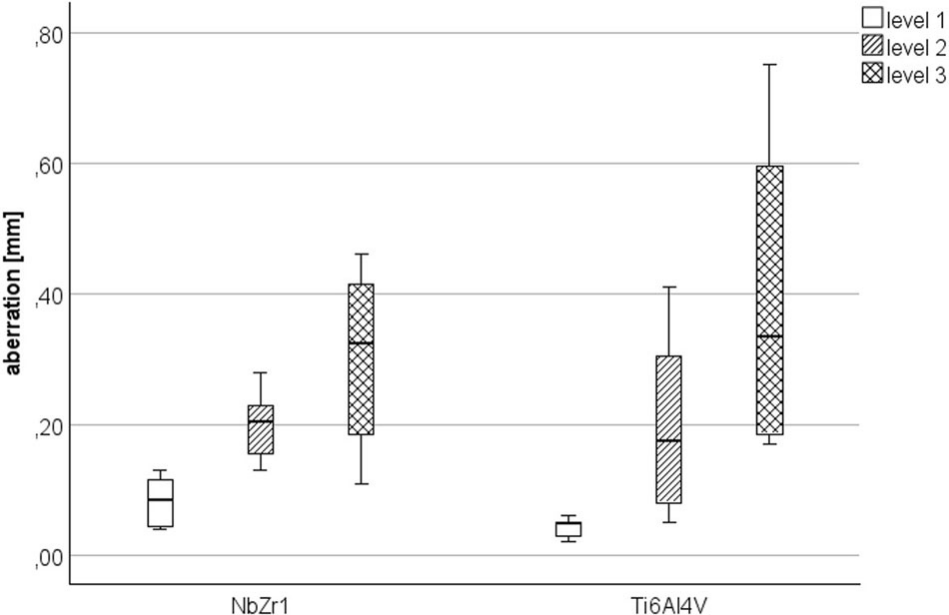
Deviation at 3 measuring levels; level 1 = implant shoulder, level 2 = lower abutment margin, level 3 = upper abutment margin

The three-dimensional evaluation of the deformation showed a minimal deviation of 0.08 ± 0.04 mm in the area of the implant shoulder at level 1 in group Nb1Zr. Higher deviations were found at levels 2 (0.19 ± 0.05 mm) and 3 (0.30 ± 0.13 mm). In the Ti6Al4V group, deviations were found at level 1 (0.04 ± 0.01 mm), level 2 (0.19 ± 0.14 mm) and level 3 (0.39 ± 0.24 mm) (Fig. 5). A comparison between both implant materials for the highest deviations at each of the three different measurement levels revealed significant differences at level 1 (*p* = 0.016), but not at level 2 (*p* = 0.981) and level 3 (*p* = 0.364). The observed deformations of the test specimens were only measured at maximum load, mainly above the implants at levels 2 and 3 on the abutment. The deviations from the initial situation were a maximum of 0.5 mm. There was no mechanical deformation of the abutment itself, only changes in position. This was caused by deformation of the abutment screws used, which ultimately led to the abutment tilting away from the implant axis. In the area of the implant shoulder on level 1, only minimal deviations of 0.08 mm occurred.

## Discussion

The aim of the presented study was to compare the load-bearing capacity of the new experimental implant made of Nb1Zr with implants of the same shape made of Ti6Al4V and to test whether the load-bearing capacity is sufficient to withstand the expected masticatory forces. The implant geometry selected here corresponds to an implant geometry that has a particularly wide range of clinical applications. The selected diameter could be used in the anterior tooth and premolar region. The implant would also be suitable for replacing individual molars.

The in-vitro tests selected here are a common method for answering this question. Multiaxial tests are particularly suitable for simulating the load during the chewing process. Chewing simulations are often combined with alternating thermal loading in order to come as close as possible to intraoral conditions. Unfortunately, a complete simulation of clinical conditions is not yet possible with modern chewing simulators [10]. In this study, the applied force of 25 N was in the lower range of the average physiological chewing force, which is between 10 N and approximately 120 N in humans during regular chewing. The individual masticatory load varies depending on the position of the tooth in the jaw and the strength of the masticatory muscles and, therefore, has different effects on the clinical success of dental implants [11]. In their 2008 paper, Rosentritt et al. have concluded that a load of 25 N has been sufficient to make an adequate prediction of clinical failure [12]. For the present study, the load during chewing simulation was set at 25 N, as aging by regular chewing was to be simulated, but the applied load should not yet lead to failure of the specimen. Overall, the artificial aging with 1 × 10^6^ cycles of chewing simulation with parallel thermocycling of 4 × 10^3^ cycles between 5 °C and 55 °C simulates an aging time of about 5 years [13].

When the test specimens were prepared, care was taken to ensure that the experimental implants were embedded in such a way that the clinical situation was reproduced as realistically as possible. Dental implants are usually placed in such a way that the area of the implant shoulder of bone-level implants is congruent with the level of the alveolar bone. Especially in the mandible, the circumferential compact bone structures can stabilize the implant shoulder against deformation. Unfortunately, however, vertical bone loss of 0.3–1.75 mm circularly around the implant often occurs during the healing phase after implant placement [14]. In the present study, the implants were embedded in the polyurethane in such a way that a distance of 2 mm to the implant shoulder remained uncontained. This simulated vertical bone loss and ensured that the implant was not protected from deformation by the embedding material.

There are two distinct experimental set-ups to for load testing: tests that are based on ISO 14801 and tests that are more related to clinical situations. In the present study, the direction and type of loading were modified to correspond to the clinical situation in the molar region. The direction of force application corresponds to the axis of clinical loading in the posterior region of the dental arch. The force was applied via an anatomically correctly shaped crown for tooth 16. In comparison the load test according to ISO 14801 maps the situation in the anterior region by angling the implant by 30° to the load direction. In addition, the load is applied via a hemispherical abutment, which results in point loading only. Compared to ISO 14801, our test arrangement also allowed lateral force application due to the resulting multipoint contact during the multiaxial chewing simulation. Although this limits comparability with studies based on ISO 14801, the data obtained is closer to clinical reality.

In some situations, such as bruxism, masticatory forces can reach up to 800 N [15]. This means that implant-supported dentures should not only withstand regular chewing forces, but also higher forces. All specimens survived artificial ageing by chewing simulation and thermocycling as expected. Accordingly, the maximum load-bearing capacity was determined for all specimens in the compression test. In the load tests, deformation of the test specimens occurred at the earliest at loads above 1114.0 N. The first hypothesis can be confirmed. Interestingly, the data collected in the present study show a relatively large standard deviation in both the Nb1Zr group and the Ti6Al4V group for both the maximum load-bearing capacity and the deformation at maximum load. This is due to the fact that the production of the test implants is not yet industrialized. Automated standard techniques for processing the experimental implant geometry do not yet exist. For the present study, each implant was manufactured individually. On the basis of the in silico load simulation, which Christiansen carried out with the experimental implant geometry also investigated here in the FE model in accordance with ISO 14801, deformation of the experimental implant in the area of the implant shoulder has been expected at a compressive load of around 550 MPa (8). In this respect, the implant tested in the present study is already similar to the implants tested in the literature. In the ISO 14801 load test, Dittmer et al. have found plastic deformation of titanium implants in the area of the implant shoulder and in the area of the implant-abutment connection on various implant systems from an applied force of approx. 400 N [16]. In their in-vitro study on the deformation of implants and abutments according to ISO 14801 published in 2023, Zhai et al. have found volume changes of about one cubic millimeter at the implant shoulder when using titanium abutments. The fracture strength after artificial aging was reported to be about 400 N for titanium implants after aging with alternating forces between 10 N and 150 N [17]. Other authors have found higher load capacities for dental implants in general. Amelya et al. have been able to show that the load-bearing capacity of implant-supported restorations is over 1000 N when the implant crowns are made of PEEK. Significantly higher loads were tolerated when the implant crowns were made of monolithic zirconia [18]. Molinero-Mourelle et al. have reported maximum load values of 1579 ± 149 N for implant-supported restorations [19]. These findings are in agreement with the results of the present study, in which the first deformations of the tested specimens were observed at a load of 1114 N and more. In line with findings by Sarafidou et al. who have also loaded implants in orthograd axis and found load capacities above 2500 N [20], the axial force application and the force distribution over the anatomically shaped occlusal surface of the zirconia crown used are partly responsible for the high load values of the implants examined in the present study underlining the importance of correct implant positioning with corresponding resulting force vectors for the long-term prognosis of implant-supported single-tooth crowns. The results of the present study show that the newly developed experimental implants made of the Nb1Zr alloy can not only withstand the expected forces during regular chewing, but can also withstand loads that are not achieved under clinical conditions.

Although dental implants have a high survival rate, mechanical complications such as deformations or screw loosening do occur. The implants examined here were able to withstand the regularly occurring masticatory forces without any problems. The second hypothesis can also be confirmed. Deformations of the test specimens were only observed at maximum load and amounted to a maximum of 0.5 mm in the area of the abutments. Only minimal deviations of 0.08 mm occurred in the area of the implant shoulder. Comparing the results with the literature, it is very likely that there was deformation or loosening of the fixing screws used for the abutment [21]. Both the data of the present study and the literature show that the main mechanical stress to be expected will be localized in the area of the implant shoulder [9, 16]. In this study, there was no mechanical deformation of the abutment itself, only positional changes. These were caused by the deformation of the abutment screws used, which ultimately caused the abutment to tilt away from the implant axis. In further studies, this assumption must be confirmed by examining the individual components of the samples examined here using SEM.

In view of the in vitro design of the study presented, a few limitations must be mentioned: the material Nb1Zr is still in the development stage and unfortunately is available only in very limited quantities, which is why only eight implants were tested. The implants are individually manufactured by hand. Accordingly, possible manufacturing differences cannot be ruled out. Furthermore, it is not yet possible to say how the load capacity will change if the abutment and screw are also made of Nb1Zr. This could be the subject of further studies.

## Conclusions

Taking into account the limitations of the present study, the following conclusions can be drawn: The load-bearing capacity of the experimental implants made of Nb1Zr does not differ from implants of the same shape made of Ti6Al4V. No complications are to be expected with the implants made of Nb1Zr under the expected masticatory load. The investigated alloy of niobium and zirconium appears to be very promising for the further development of the experimental drug releasing implant.
